# Hemodynamic Response to High- and Low-Load Resistance Exercise in Patients with Coronary Artery Disease: A Randomized, Crossover Clinical Trial

**DOI:** 10.3390/ijerph18083905

**Published:** 2021-04-08

**Authors:** Tim Kambic, Vedran Hadžić, Mitja Lainscak

**Affiliations:** 1 Department of Research and Education, General Hospital Murska Sobota, 9000 Murska Sobota, Slovenia; tim.kambic@gmail.com; 2 Faculty of Sport, University of Ljubljana, 1000 Ljubljana, Slovenia; vedran.hadzic@fsp.uni-lj.si; 3 Division of Cardiology, General Hospital Murska Sobota, 9000 Murska Sobota, Slovenia; 4 Faculty of Medicine, University of Ljubljana, 1000 Ljubljana, Slovenia; 5 Faculty of Natural Sciences and Mathematics, University of Maribor, 2000 Maribor, Slovenia

**Keywords:** cardiac rehabilitation, resistance training, blood pressure, heart rate

## Abstract

Low-load resistance exercise (LL-RE) is recommended as an adjunct therapy to aerobic exercise during cardiac rehabilitation in patients with coronary artery disease. The safety and hemodynamic response to high-load (HL) RE remain unknown. The aim of this study was to evaluate the hemodynamic response during both HL-RE and LL-RE prior to cardiac rehabilitation. Forty-three patients with coronary artery disease and/or percutaneous coronary intervention performed three sets of leg-press exercise using HL-RE (eight repetitions at the intensity of 80% of one repetition maximum (1-RM)) and LL-RE (16 repetitions at the intensity of 40% 1-RM) in a randomized crossover sequence. Heart rate (HR), systolic blood pressure (SBP), diastolic blood pressure (DBP), and rating of perceived exertion were measured at baseline, after each set of RE and post-exercise. No clinically relevant changes in HR and BP or in patient-reported symptoms were recorded during HL-RE or LL-RE. Compared with baseline, HR and SBP increased during LL-RE (from 66 bpm to 86 bpm, time effect: *p* < 0.001; from 129 mmHg to 146 mmHg, time effect: *p* < 0.001) and HL-RE (from 68 bpm to 86 bpm, time effect: *p* < 0.001; from 130 mmHg to 146 mmHg, time effect: *p* < 0.001). Compared with HL-RE, the increase in HR was greater after the final set of LL-RE (32% vs. 28%, *p* = 0.015), without significant differences in SBP and DBP between LL-RE and HL-RE. Rating of perceived exertion was higher after the 1st set of HL-RE compared with LL-RE (median (interquartile range): 6 (5–7) vs. 6 (5–6), *p* = 0.010). In patients with coronary artery disease, both HL-RE and LL-RE were safe and well-tolerated. Hemodynamic changes were similar and within the physiological response to RE.

## 1. Introduction

Cardiac rehabilitation (CR) is a recommended multidisciplinary intervention for the treatment and secondary prevention of cardiovascular diseases, with emphasis on improving patients’ cardiovascular risk factors, exercise capacity, nutritional status, and psychosocial well-being [1]. Exercise training represents a core component of CR [1,2], in which aerobic training is predominately prescribed and studied, whereas resistance training remains underused [3]. Despite being recommended for >20 years [1,4,5] the implementation of resistance training in clinical practice is still limited by the heterogeneous training protocols, including variation of intensity (30–80% of one repetition maximum (1-RM)) [1,2,5,6], number of repetitions (8–20) [1,2,5], rest periods between sets (30–90 s), and speed of repetitions (1–3 s per contraction) [7,8]. There also remains a concern about excessive changes in blood pressure (BP) and heart rate (HR), in particular when the patient is exercising with the Valsalva maneuver [6,9,10]. All these issues reflect the lack of consensus between scientific organizations in different countries [3,11].

Only a few mechanistic studies have demonstrated the potential safety of high-load (HL) compared with currently established low-load (LL) resistance exercise (RE) training in patients with coronary artery disease (CAD) [7,8,12,13], patients with hypertension [14], and in young [15] and older adults [16,17]. Two studies in patients with CAD have compared acute hemodynamic response (measured as HR, BP, and cardiac output changes compared with baseline) during HL-RE (70–90% 1-RM, 4–10 repetitions) and LL-RE (30–40% 1-RM, 15–20 repetitions) [7,13]. Both studies showed greater increase in HR, BP, cardiac output, and rating of perceived exertion (RPE) during LL-RE [7,13], without any adverse symptoms or events (e.g., dizziness, chest pain, loss of consciousness, shortness of breath, muscle pain). Breaks between sets varied from 1 to 4 min in both studies [8,13]. In addition to the intensity of RE, a study also demonstrated the importance of the speed of repetitions and duration of breaks between sets on the hemodynamic response. This study reported a lower hemodynamic increase during three sets of 10 repetitions at an intensity of 70% of 1-RM, with a faster speed of repetitions (1 s of concentric and 1 s of eccentric contraction) and longer breaks of 90 s between sets [7]. It appears, therefore, that the duration of workload within a set, and length of breaks between sets, may be important in the hemodynamic response and patients’ perceived exertion. However, the effects of exercise intensity cannot be ruled out, as previous studies have failed to balance the exercise volume between LL-RE and HL-RE [8,13].

Acute hemodynamic response to RE has been studied only in CAD patients with previous training experience in CR (<1 to 6 months of engagement) [7,8,13], where exercise training may have had an impact on hemodynamic adaptations [6] and lowered the RPE. To date, no study has examined the effects of different RE loads on the hemodynamic response during the entry phase to CR (<1 week). The aim of our study, therefore, was to examine the hemodynamic response to LL-RE and HL-RE in patients with CAD at the start of their CR.

## 2. Materials and Methods

### 2.1. Study Design

**Figure 1:**
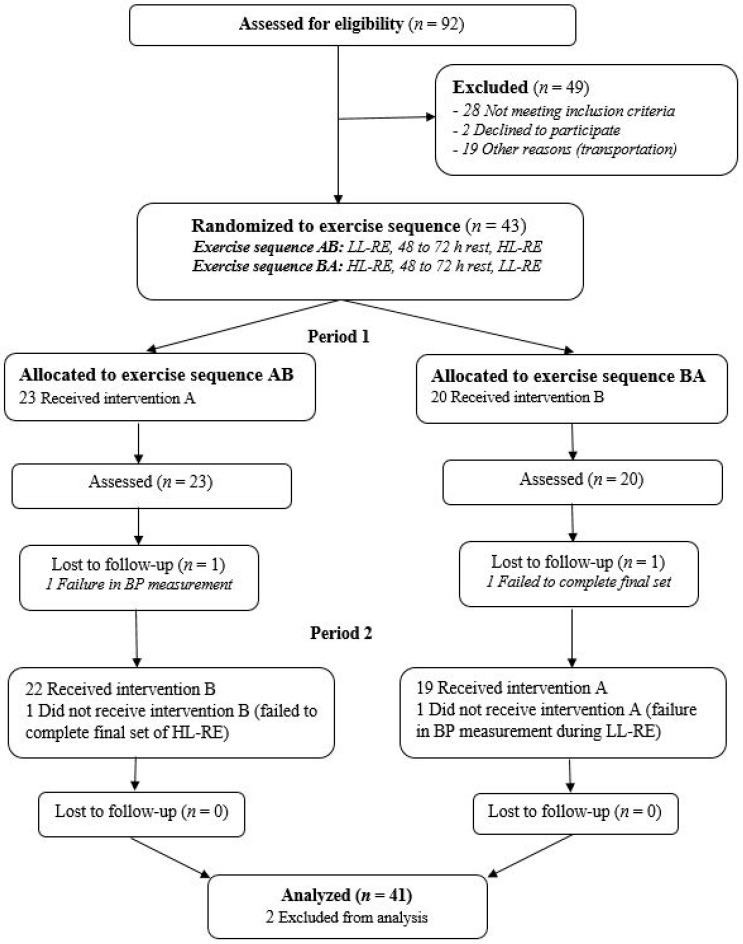
CONSORT flow chart of the study. LL, low load; HL, high load; RE, resistance exercise; BP, blood pressure.

The study was designed as a randomized, crossover clinical trial in line with CONSORT guidelines (Figure 1) [18]. After initial ambulatory screening by a cardiologist and a cardiopulmonary exercise test, patients were randomized into an AB exercise sequence (LL-RE, 48–72 h of rest, HL-RE) or a BA exercise sequence (HL-RE, 48–72 h of rest, LL-RE) using a 1:1 computer-generated randomization ratio. Measurements were made during patients’ first three visits to outpatient CR. During the first visit, they were familiarized with correct lifting and breathing techniques, and performed a 1-RM test on a leg-press machine. During the second and third visits, the hemodynamic response to RE was measured. Patients had a rest of 48–72 h between measurement days.

### 2.2. Participants

Patients with stable CAD (≥1 month after acute coronary syndrome and/or percutaneous coronary intervention) were recruited from the Division of Cardiology at General Hospital Murska Sobota and enrolled during an ambulatory visit after discharge. Inclusion criteria were as follows: age 18–85 years, documented CAD, left ventricular ejection fraction ≥40%, and completion of a cardiopulmonary exercise test [1]. Exclusion criteria were adopted from previous guidelines [6,19]. Before enrollment, all patients were informed about the methods, procedures, and potential risks during the study, and were asked to give their written consent. The study design was approved by the National Medical Ethics Committee (registration date: 15 June 2020; registration number: 0120-573/2019/15) and is registered with ClinicalTrials.gov (accessed on 29 October 2020, identifier: NCT04638764).

### 2.3. Measurement of Maximal Aerobic Capacity

Maximal aerobic capacity was measured using an adjusted ramp protocol [20] on a Schiller ERR 911 ergometer bicycle (Schiller, Baar, Switzerland) and a Cardiovit CS-200 Excellence Ergo-Spiro system (Schiller, Baar, Switzerland). Patients performed two repetitions of spirometry, followed by 3 min rest to determine baseline BP, HR, and gas exchange. The test started with patients cycling without a workload for 3 min, followed by an increase every minute for an additional 10–25 W until exhaustion or any relevant reason to stop testing (e.g., chest pain). The supervising nurse followed any potential signs or symptom-limited indications for exercise termination, as recommended by the American Heart Association [20].

### 2.4. Measurement of Maximal Strength

A Life Fitness Leg Press Pro 2 machine (Life fitness Inc., Rosemont, IL, USA) was used for leg-strength testing. Before measurement, each patient performed a general warm up (5 min cycling at 50% of maximum HR with cadence between 50 and 60 rpm and dynamic stretching of lower limbs), followed by familiarization with the machine to learn correct lifting and breathing techniques (exhalation during concentric contraction to avoid the Valsalva maneuver [10]). During the test, patients were first instructed to complete a warm-up set comprising eight repetitions at 50% and six repetitions at 70% of their perceived 1-RM. The load was then progressively increased until reaching the workload that could be lifted between three and five times (3–5 RM), with 2–3 min rest between efforts [21,22]. The 1-RM was calculated using a well-established 1-RM prediction equation (Equation (1)) [23]. The submaximal assessment of 1-RM (3–5 RM) is highly correlated with the actual 1-RM test [24], and presents a safe alternative to the actual 1-RM test in unexperienced, medically supervised patients [22].

predicted 1-RM = maximal load lifted/1.0278–0.0278 × number of repetitions(1)

### 2.5. Measurement of Hemodynamic Response during Resistance Exercise

Hemodynamic response included measurements of HR, BP, and blood oxygen saturation during RE. We used an OMRON HBP 1320 professional BP monitor (Omron Healthcare, Inc., Vernon Hills, IL, USA) [20,25] and a Nellcor Oximax N-65 pulse oximeter (Covidien LLC, Manfield, MA, USA). RPE was evaluated using a short version of Borgs’ scale (0–10) [26]. Hemodynamic parameters were measured in a seated position at baseline (3 min before exercise), then after the final repetition of each set, and again 3 min post-exercise. After a general warm-up and baseline hemodynamic measurements, patients performed exercises according to the sequence of randomization. The exercises consisted of three sets of either 16 repetitions at 40% of 1-RM (LL-RE) or eight repetitions at 80% of 1-RM (HL-RE), with a lifting cadence ratio of 1 s of concentric contraction and 1 s of eccentric contraction, and with 90 s of rest between sets [7,8,22]. Such training parameters were previously shown to induce a smaller increase in HR, BP, and cardiac output compared with slower repetition speeds and/or shorter breaks between sets [7]. The cumulative load (kg) was balanced between both types of RE, according to maximal repetitions allowed in the HL-RE (eight repetitions at 80% of 1-RM) to eliminate the potential effects of training load [22]. Thus, we equated the training load in both types of RE using Equation (2). The same RE protocol using other resistance loads was performed after 48–72 h rest.

cumulative load = 8 repetitions × load at 80% of 1-RM = 16 repetitions × load at 40% of 1-RM(2)

### 2.6. Statistical Analysis

Categorical variables are presented as frequencies and percentages; numeric variables are presented as means and standard deviations for normally distributed variables, or as medians and interquartile ranges for asymmetrically distributed variables. Numeric variables were screened for the normality of distribution (using the Shapiro–Wilk test), homogeneity of variances (Levene’s test), and sphericity (Mauchly’s test), where appropriate. Two-way analysis of variance (ANOVA) was used to compare the main effects of time (baseline, during exercise, and post-exercise measurements), load (HL-RE vs. LL-RE), and interaction load × time, and the effect sizes were calculated for each variable (partial eta squared) [27]. We additionally used the Bonferonni correction for multiple comparisons to examine differences in hemodynamic response between both types of RE at a given time point or hemodynamic response during HL-RE and LL-RE. IBM SPSS 25 software (SPSS Inc., Armonk, NY, USA) was used for statistical analyses at the level of significance (*p*-value) <0.05.

## 3. Results

Ninety-two patients were evaluated for CR eligibility and 43 were enrolled. Baseline characteristics (mean (SD)) of the patients were: age = 61 (10) years; height = 172.3 (7.8) cm; weight = 88.09 (16.66) kg; and left ventricular ejection fraction = 53% (10%). Other anthropometric variables, clinical data, and exercise performance variables are shown in Table 1.

Twenty-three patients were randomized to exercise sequence AB and 20 patients to sequence BA (Figure 1). All patients underwent their first RE session in Period 1. In exercise sequence AB, one patient was lost to follow-up because of a failed BP measurement after the first set of exercises, and one patient failed to complete the third set of HL-RE (exercise sequence BA) because of muscle weakness. Finally, 41 patients with complete data from Periods 1 and 2 were included in the analysis.

No major cardiovascular (palpitations, atrial fibrillation, arrhythmias, chest pain, BP > 220/110 mmHg, etc.) or musculoskeletal complications were reported during or after LL-RE and HL-RE. During the exercises, three (7%) patients reported a few seconds of lightheadedness, but without vertigo or loss of consciousness, after each set of HL-RE. After the second and third sets of LL-RE, some patients reported muscle fatigue (three; 7.0%) and shortness of breath (four; 9.3%) that terminated before the start of the next set. The following ranges were measured during LL-RE and HL-RE, respectively: HR: +26 bpm and +37 bpm; systolic blood pressure (SBP): −16 to +53 mmHg and −10 to +52 mmHg; diastolic blood pressure (DBP): −23 to +24 mmHg and −15 to +17 mmHg. SBP decreased in patients who reported lightheadedness.

**Figure 2:**
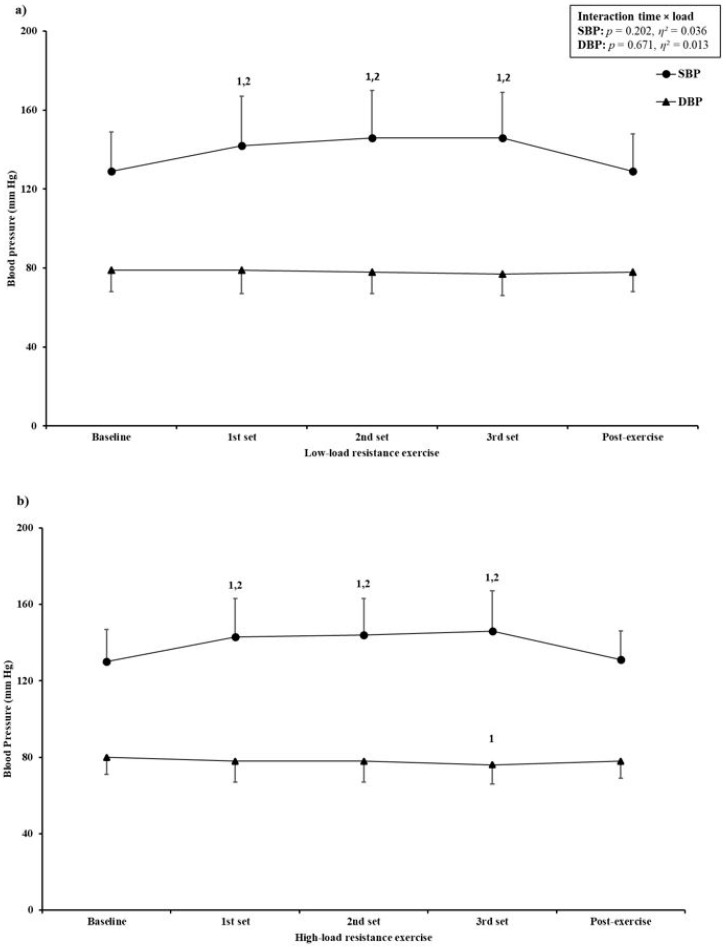
Blood pressure response to (**a**) low-load resistance exercise and (**b**) high-load resistance exercise. SBP: systolic blood pressure; DBP: diastolic blood pressure; η²: partial eta squared. Data are expressed as mean (dots and triangles) and SD (vertical lines). 1 = significantly different from baseline; 2 = significantly different from post-exercise.

During the both types of RE, there was a significant increase in HR (using BP monitor, time effect: *p* < 0.001; using oximeter, time effect: *p* < 0.001), SBP (time effect: *p* < 0.001), blood oxygen saturation (time effect: *p* = 0.002), and RPE (time effect: *p* < 0.001). Diastolic BP significantly decreased during both types of RE (time effect: *p* < 0.001) (Figure 2, Table 2). Compared with baseline and post-exercise, HR and SBP were significantly higher after the first set (*p* < 0.001 for HR and SBP in both types of RE), second set (*p* < 0.001 for HR and SBP in both types of RE), and third set (*p* < 0.001 for HR and SBP in both types of RE), with no differences between sets for either hemodynamic parameter. Furthermore, when HR was measured using an oximeter, the value was significantly higher after the third set compared with the second (*p* = 0.037) and first set (*p* < 0.001) of LL-RE. Diastolic BP remained unchanged during LL-RE; however, during HL-RE there was a significant decrease after the third set compared with baseline (−4 mmHg, *p* = 0.011). Blood oxygen saturation changed significantly during RE (Table 2). After LL-RE there was a significant increase in blood oxygen saturation compared with baseline (+1%, *p* = 0.010), whereas after HL-RE the value remained unchanged. With the exception of significant time × load interaction for blood oxygen saturation (*p* = 0.026), there was no significant interaction for HR (HR, *p* = 0.353; HR oxi, *p* = 0.099), SBP (*p* = 0.202), DBP (*p* = 0.671), and RPE (*p* = 0.323).

Compared with baseline, both types of RE induced similar increases of HR (measured with BP monitor; interaction, *p* = 0.318), SBP (interaction, *p* = 0.112), and DBP (interaction, *p* = 0.933) after each set of exercises and post-exercise (Table 3). However, when HR was measured using a pulse oximeter, there was a significant interaction (*p* = 0.019). After the third set of LL-RE, there was greater increase in HR compared with HL-RE (32% vs. 28%, *p* = 0.015) when the HR was measured using an oximeter.

Perceived exertion was rated with the minimum grade at baseline and at post-exercise on every occasion; thus, we compared only the differences after all three sets of RE (Table 2). RPE significantly increased during both types of RE (time effect, *p* < 0.001); however there was no significant time × load interaction (*p* = 0.323). During the LL-RE, there was a significantly higher RPE after the third (*p* < 0.001) and second sets (*p* < 0.001) compared with the first set, whereas during HL-RE the RPE increased significantly after each set of RE. After the first set of HL-RE, patients rated the exertion significantly higher than after LL-RE (6 [5, 7] vs. 6 [5, 6], *p* = 0.010), with no differences between loads in the second and third sets.

## 4. Discussion

In our study, both HL- and LL-RE were shown to be generally safe, feasible, and well-tolerated in patients with CAD. Compared with baseline, all hemodynamic parameters increased during the exercises and returned to baseline levels after both HL-RE and LL-RE. HL-RE induced similar increases to LL-RE in all hemodynamic parameters, whereas exertion was rated higher only after the first set of HL-RE when compared with LL-RE.

RE is historically associated with BP changes that can cause patient symptoms and even loss of consciousness. This may have interfered with RE delivery in CR programs. Studies to assess safety aspects and response to RE in patients with CAD are few [7,8,12,13], and ours is a relevant addition to the literature. As previously reported, we did not detect any signals for safety concerns, as variations in vital signs were within expected ranges and patients experienced no relevant symptoms.

Since the early studies on hemodynamic responses during resistance training in bodybuilders [28] and CAD patients [12], little progress has been made to better understand the cardiovascular response to different loads of RE and training in patients with cardiovascular disease [9]. Nevertheless, two studies demonstrated a higher increase in BP and HR during LL-RE compared with HL-RE in patients with CAD [8,13]. In addition, HR and SBP were the highest in the final (third or fourth) sets in those studies when compared with baseline and the first set after LL-RE [8,13]. Similar results have been obtained in healthy adults [15], the elderly [16], and patients with hypertension [14]. These findings are in contrast to ours, where we demonstrated similar hemodynamic responses between exercise loads. These differences can be partially explained by the level of training and maximal muscle strength. In the past, studies have enrolled already trained patients with CAD (from >1 to 6 months of training) [8,13], although those patients had lower muscle strength compared with our sample. Most studies have not reported patients’ maximal leg strength; however, one study reported lower maximal strength of the patients (mean submaximal strength of leg extensors, 4-RM = 38 kg; 15-RM = 24 kg; 1-RM ≅ 44 kg) [13], compared with our sample measured on the leg-press machine (132 (48 kg)). The lower maximal strength profile of the patients in the previous study [13] might have led to increased activity of the exercise pressor reflex, which is modulated by an increase firing rate of group III and IV afferents in the lower limbs (as a response to higher mechanical and metabolic activity in the exercising limbs), and thus signaled the central command on a basal level to increase sympathetic activity during the exercise [29]. This may have resulted in an acutely increased BP, as observed in previous studies, especially during longer duration of LL-RE (greater metabolic response in knee extensors) [8,13]. As the majority of (lifelong) CR programs are based on aerobic training only, with an occasional addition of some forms of calisthenics [2,3], this may additionally confirm our postulations about the importance of maximal strength in this patient group.

As previously reported, LL-RE induced a greater increase in HR (69%, from 67 bpm to 113 bpm) compared with HL-RE (39%, from 64 bpm to 89 bpm) [13]. We report similar changes but to a lesser degree. This could be due to a different submaximal intensity of RE (4 RM = 87% ± 2% of 1-RM; 15 RM = 55% ± 5% of 1-RM) [13] than used in our study. Several previous studies in patients with CAD [7,8] emphasize the important effects of exercise duration and rests between sets on the hemodynamic response. In one methodological study, a greater increase in hemodynamic parameters was demonstrated when the repetitions were made at a slow (3 s/3 s of concentric and eccentric contraction) or moderately slow pace (2 s/2 s) compared with a faster pace (1 s/1 s), and when the rest period between sets was >60 s [7]. The same authors reported a similar response in HR (increase from 78 bpm after the first set to 81 bpm after the third) and SBP (increase from 146 mmHg after the first set to 160 mmHg in the third), when using the same speed of repetitions (1 s/1 s) and rest period (90 s) between sets [7] as in our study. In line with findings in patients with CAD, several previous studies enrolling healthy young adults [15] and elderly with and without hypertension [14,16] reported higher HR and BP during longer sets of LL-RE (>20 repetitions per set or set to voluntary exhaustion) compared to shorter sets of HL-RE (4–9 repetitions) with shorter breaks between sets (<60 s). Generally, when similar LL-RE (4-RM) and HL-RE (15–20 RM) protocols were performed, there was no difference in hemodynamic response to RE between healthy young adults [15] and patients with CAD [13]. In contrast, one study reported higher hemodynamic response to HL-RE and LL-RE in elderly subjects with hypertension compared to their healthy peers [14]. However, it must be noted that the authors did not adjust the analysis of hemodynamic response based on group differences in baseline BP. However, with aging and age-related cardiovascular comorbidities (e.g., hypertension) likely contributing to decreased arterial compliance and arterial wall elasticity [30], such differences between healthy adults and the elderly are expected. Additional studies comparing the hemodynamic response in healthy, elderly, and clinical populations are warranted.

The intensity of the RE should be followed using the Borg’s scale (6–20 or 0–10) [26], and patients with cardiovascular disease are recommended not to exceed a fairly light to somewhat hard exercise level [1,6]. Most hemodynamic studies [7,8,14] did not report the RPE after each set or during the RE, while several reported conflicting results [13,16]. Partly in line with our findings, no difference in RPE during LL-RE and HL-RE has been reported for healthy adults [16]. Limited literature is available on patients with CAD; however, significantly higher RPE during LL-RE has been reported, but those CAD patients underwent testing at higher intensities of LL-RE and HL-RE [13].

To the best of our knowledge, ours is the first study to examine the hemodynamic response to HL-RE and LL-RE when these are included in a CR program. Formal calculation of sample size was not possible in our study, but we believe that with 41 patients it was adequately powered, particularly when compared with previous literature [8,9,13,29]. Hemodynamic methodology deserves some attention, as we did not use beat-to-beat measurements, as has been done in some previous reports [7,8,13]. Nevertheless, even in the absence of correlation studies, we would argue that the methodology as used in our study replicates the standard procedures during CR, which makes it applicable to current clinical practice.

## 5. Conclusions

Our study has demonstrated that RE as a part of CR is safe, and that different loads (LL vs. HL) elicit similar physiological responses in HR and BP. Patients did not experience any relevant difficulties with either type of RE, and these elicited similar RPE in the second and third sets of RE. Future studies should compare routinely used oscillometric methods with continuous monitoring of hemodynamic responses during RE in patients with CAD, as well as in patients with other cardiovascular diseases, such as heart failure, who could benefit from RE as a part of their CR.

## Figures and Tables

**Table 1:** Demographics, comorbidities, pharmacological therapy, cardiovascular risk factors, and exercise performance.

Anthropometrics	M (SD)
Height (cm)	172.3 (7.8)
Weight (kg)	88.09 (16.66)
Waist circumference (cm)	105.3 (12.6)
Hip circumference (cm)	106.1 (7.6)
**Clinical Data**	**M (SD) or Me (Q1, Q3)**
LVEF (%)	53 (10)
Time from clinical event to inclusion in CR (months)	2.0 (1.5, 2.0)
**Myocardial Infarction**	**f (%)**
NSTEMI	20 (46.51)
STEMI	20 (46.51)
Unstable AP	3 (6.98)
**Comorbidities and Risk Factors**	**f (%)**
Arterial hypertension	31 (72.09)
Hyperlipidemia	35 (81.40)
Diabetes	6 (13.95)
Atrial fibrillation	3 (6.989)
Thyroid disease	4 (9.30)
Renal disease	3 (6.98)
**Smoking**	**f (%)**
Non-smoker	14 (32.56)
Ex-smoker	21 (48.84)
Smoker	8 (18.60)
**Pharmacological Therapy**	**f (%)**
Aspirin	41 (95.35)
Beta blocker	43 (100.00)
ACE inhibitor/ARB	41 (95.35)
Statin	43 (100.00)
Antiplatelet drug	42 (97.67)
Anticoagulation drug	2 (4.65)
Diuretic	5 (11.63)
**Exercise Characteristics**	
**Aerobic Capacity**	**M (SD) or Me (Q1, Q3)**
Power output (W)	128 (45)
VO_2_ max (L/min)	1.58 (0.54)
VCO_2_ max (L/min)	1.69 (0.63)
VO_2_ max (mL/kg/min)	18.10 (5.27)
Respiratory exchange ratio	1.05 (0.09)
Ve/VCO_2_ slope	28.22 (26.28, 31.85)
**Maximal Leg Strength, M (SD)**	**M (SD) or Me (Q1, Q3)**
Submaximal 1-RM	122 (45)
Number of reps at submaximal 1-RM	4 (3,5)
Calculated 1-RM (kg)	132 (48)
40% 1-RM (kg)	53 (19)
80% 1-RM (kg)	105 (38)

Me (Q1, Q3), median (first quartile, third quartile); LVEF: left ventricular ejection fraction; (N)STEMI: (non-) ST segment-elevated myocardial infarction; AP: angina pectoris; ACE: angiotensin-converting-enzyme; ARB: angiotensin II receptor blockers; VO_2_: maximal oxygen consumption; Ve/VCO_2_: ventilation/carbon dioxide production ratio; 1-RM: one-repetition maximum.

**Table 2:** Hemodynamic response and rating of perceived exertion at baseline, during, and after LL-RE and HL-RE.

Parameter Load		Baseline	1st set	2nd Set	3rd Set	Post Exercise	Interaction Time × Load *p* (η^2^)
HR (bpm)	40% 1-RM	66 (9)	81 (11) ^a,b^	83 (11) ^a,b^	83 (12) ^a,b^	67 (9)	0.353 (0.027)
	80% 1-RM	68 (10)	84 (13) ^a,b^	84 (13) ^a,b^	84 (13) ^a,b^	69 (10)	
HR oxi. (bpm)	40% 1-RM	66 (9)	83 (10) ^a,b^	85 (11) ^a,b^	86 (11) ^a,b,c,d^	67 (10)	0.099 (0.050)
	80% 1-RM	68 (10)	86 (13) ^a,b^	86 (13) ^a,b^	86 (13) ^a,b^	68 (10)	
Sa (%)	40% 1-RM	97 (96, 97)	97 (96, 98)	97 (96, 98)	97 (97, 98)	98 (97, 98) ^a^	0.026 (0.072)
	80% 1-RM	97 (96, 98)	97 (97, 98)	98 (97, 99)	98 (97, 99)	97 (96, 98)	
RPE (grade)	40% 1-RM	-	6 (5, 6)	6 (5, 7) ^c^	7 (5, 8) ^c^	-	0.323 (0.027)
	80% 1-RM	-	6 (5, 7)	7 (6, 7) ^c^	7 (6, 8) ^c,d^	-	

Data are expressed as mean (SD) or as median (Q1, Q3). η^2^: partial eta squared; HR: heart rate; oxi.: pulse oximeter; Sa: blood oxygen saturation; RPE: rating of perceived exertion; 1-RM: one-repetition maximum. ^a^ Significantly different compared with baseline; ^b^ significantly different compared with post-exercise; ^c^ significantly different compared with first set; ^d^ significantly different compared with second set.

**Table 3:** Baseline percentage change of hemodynamic parameters during low-load (LL) resistance exercise (RE) and high load (HL)-RE.

Parameter Load		Δ First Set vs. Baseline	Δ Second Set vs. Baseline	Δ Third Set vs. Baseline	Δ Post-Exercise vs. Baseline	Interaction Time × Load *p* (η^2^)
HR (%)	40% 1-RM	23 (10)	25 (10)	25 (10)	1 (−1, 4)	0.318 (0.029)
	80% 1-RM	24 (12)	24 (12)	25 (12)	1 (−2, 4)	
HR oxi. (%)	40% 1-RM	26 (20, 38)	29 (10)	32 (13)	2 (−3, 4)	0.019 (0.080)
	80% 1-RM	25 (18, 34)	28 (11)	28 (11) *	2 (−3, 4)	
SBP (%)	40% 1-RM	10 (8)	11 (9)	12 (8, 18)	0 (6)	0.112 (0.048)
	80% 1-RM	10 (9)	13 (11)	11 (6,18)	1 (6)	
DBP (%)	40% 1-RM	0 (−3, 4)	−1 (−7, 2)	−2 (7)	0 (−4, 2)	0.933 (0.004)
	80% 1-RM	−1 (5, 2)	−3 (−7, 3)	−4 (8)	−2 (−6, 1)	

Data are expressed as mean (SD) or as median (Q1, Q3). η^2^: partial eta squared; Δ: percentage change; HR: heart rate; oxi.: pulse oximeter; SBP/DBP: systolic/diastolic blood pressure. * Significantly different compared to LL-RE.

## Data Availability

The supporting data for this study are available from the corresponding author upon reasonable request.
